# Functional and Neuroprotective Role of Striatal Adenosine A_2A_ Receptor Heterotetramers

**DOI:** 10.1089/caff.2019.0008

**Published:** 2019-09-17

**Authors:** Sergi Ferré, Francisco Ciruela

**Affiliations:** ^1^ Integrative Neurobiology Section, National Institute on Drug Abuse, Intramural Research Program, National Institutes of Health, Baltimore, Maryland.; ^2^ Pharmacology Unit, Department of Pathology and Experimental Therapeutics, School of Medicine, IDIBELL, University of Barcelona, L'Hospitalet de Llobregat, Barcelona, Spain.; ^3^ Institute of Neurosciences, University of Barcelona, Barcelona, Spain.

**Keywords:** adenosine A_2A_ receptor, adenosine A_1_ receptor, dopamine D_2_ receptor, GPCR heteromers, Parkinson's disease, restless legs syndrome

## Abstract

In the striatum, adenosine A_2A_ receptors (A_2A_R) are mainly expressed within the soma and dendrites of the striatopallidal neuron. A predominant proportion of these striatal postsynaptic A_2A_R form part of the macromolecular complexes that include A_2A_R-dopamine D_2_ receptor (D_2_R) heteromers, G_olf_ and G_i/o_ proteins, and the effector adenylyl cyclase (AC), subtype AC5. The A_2A_R-D_2_R heteromers have a tetrameric structure, constituted by A_2A_R and D_2_R homomers. By means of reciprocal antagonistic allosteric interactions and antagonistic interactions at the effector level between adenosine and dopamine, the A_2A_R-D_2_R heterotetramer-AC5 complex acts an integrative molecular device, which determines a switch between the adenosine-facilitated activation and the dopamine-facilitated inhibition of the striatopallidal neuron. Striatal adenosine also plays an important presynaptic modulatory role, driving the function of corticostriatal terminals. This control is mediated by adenosine A_1_ receptors (A_1_R) and A_2A_R, which establish intermolecular interactions forming A_1_R-A_2A_R heterotetramers. Here, we review the functional role of both presynaptic and postsynaptic striatal A_2A_R heterotetramers as well as their possible neuroprotective role. We hypothesize that alterations in the homomer/heteromer stoichiometry (i.e., increase or decrease in the proportion of A_2A_R forming homomers or heteromers) are pathogenetically involved in neurological disorders, specifically in Parkinson's disease and restless legs syndrome.

## Introduction: Precoupling and Oligomerization

Precoupling of G protein-coupled receptors (GPCR) with G proteins and signaling molecules and receptor oligomerization are two concepts that are changing our classical views of GPCR physiology and pharmacology.^1,2^ The classical view of freely moving molecules of GPCR, G proteins, and effectors in the plasma membrane, which establish ligand-guided associations and dissociations by random collision (collision-coupling mode), is being replaced by a ligand-induced rearrangement of precoupled molecules.^1^ Furthermore, the classical view of single GPCR units is being replaced by oligomeric functional units. A large number of experimental data support the view of a common functional building block constituted by two GPCR units (homodimer) and one heterotrimeric G protein (with its preferred α and βγ subunits).^3^

A GPCR heteromer is defined as “a macromolecular complex composed of at least two (functional) receptor units (protomers) with biochemical properties that are demonstrably different from those of its individual components.”^4^ It is becoming apparent that a common quaternary structure of GPCR heteromers is a heterotetramer, formed by two different GPCR homodimers coupled to their cognate G proteins.^1,3^ This has been recently reported as the most probable quaternary structure of two striatal adenosine A_2A_ receptor (A_2A_R) heteromers, the postsynaptic A_2A_R-dopamine D_2_ receptor (D_2_R) heterotetramer and the presynaptic adenosine A_1_ receptor (A_1_R)-A_2A_R heterotetramer.^4,5^ Here, we review the functional role of both heterotetramers as well as their possible neuroprotective role. Thus, we hypothesize that alterations in the homomer/heteromer stoichiometry (i.e., increase or decrease in the proportion of GPCR protomers forming homomers or heteromers) are pathogenetically involved in neurological disorders, specifically in Parkinson's disease (PD) and restless legs syndrome (RLS).

## Functional Role of the Postsynaptic Striatal A_2A_R-D_2_R Heterotetramer

**FIG. 1.**
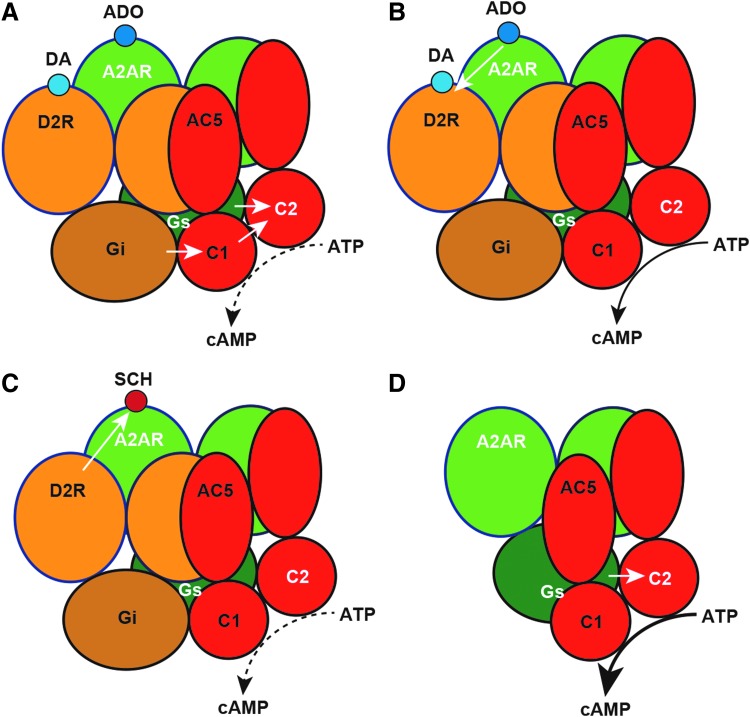
Functional and pharmacological properties of the A_2A_R-D_2_R heterotetramer. **(A)** Canonical interaction, by which a D_2_R agonist, such as DA, counteracts the effect of an A_2A_R agonist, such as ADO, by a Gs-Gi antagonistic interaction at the AC level (subtype AC5). (**B)** Allosteric interaction, by which A_2A_R ligands antagonistically counteract the affinity and efficacy of D_2_R ligands. **(C)** Ligand-independent changes in the properties of A_2A_R ligands on heteromerization with the D_2_R, such as the selective decrease in the affinity of the A_2A_R antagonist SCH442416. **(D)** Increased A_2A_R signaling in the absence of the D_2_R and in the absence of A_2A_R ligands (constitutive activity) by the A_2A_R homomer. C1 and C2, catalytic domains of AC5. *White arrows* indicate the direction of the intermolecular interaction. *Black arrows* indicate the intensity of cAMP formation (from *broken* to *small* and *large solid arrows*). A_2A_R, A_2A_ receptors; AC, adenylyl cyclase; ADO, adenosine; D_2_R, D_2_ receptor; DA, dopamine.

In the striatum, A_2A_R and D_2_R are mostly expressed within the soma and dendrites of the striatopallidal neuron, one of the two subtypes of GABAergic efferent neurons that constitute more than 95% of the striatal neuronal population.^6^ A predominant proportion of these striatal postsynaptic A_2A_R and D_2_R form part of the macromolecular complexes that include A_2A_R-D_2_R heterotetramers, G_s_ (more properly G_olf_ subtype, but G_s/olf_ proteins are referred as G_s_ proteins, for short) and G_i/o_ proteins (G_i_ proteins, for short), and the effector adenylyl cyclase (AC) subtype AC5 (Fig. 1).^5,7^ These complexes provide the frame for the canonical G_s_-G_i_ antagonistic interaction at the AC level (Fig. 1A). This canonical interaction implies the ability of an activated G_i_-coupled receptor to inhibit a G_s_-coupled receptor-mediated AC activation,^8^ thus requiring the simultaneous respective interaction of the Ras GTPase domains of the α subunits of the G_s_ and G_i_ proteins with the C2 and C1 catalytic domains of AC5.^9^ For instance, through this interaction, activation of D_2_R leads to an inhibition of A_2A_R-mediated activation of AC (Fig. 1A). In addition, the precise quaternary structure of the A_2A_R-D_2_R heterotetramer provides the frame for the ability of A_2A_R ligands to establish allosteric interactions with D_2_R ligands, not only between agonists but also between antagonists.^10^ When either an A_2A_R agonist or an A_2A_R antagonist binds to the orthosteric sites of the A_2A_R homodimer within the A_2A_R-D_2_R heterotetramer, they both produce an allosteric decrease in the affinity and efficacy of D_2_R ligands (Fig. 1B). On the contrary, when an A_2A_R agonist and an A_2A_R antagonist bind simultaneously to the two orthosteric sites of the A_2A_R homodimer, they counteract each other's effects.^10^

Furthermore, there is also evidence for significant reciprocal allosteric interactions, by which D_2_R agonists negatively influence A_2A_R agonist binding.^11,12^ This would be expected by the experimentally supported evidence that indicates that, according to Kenakin, “the allosteric energy flow is reciprocal in nature; that is, if the allosteric modulator changes the affinity of the agonist, then the agonist will also change the affinity of the modulator in a like manner.”^13^ Different efficacies where observed when evaluating the negative allosteric modulation of A_2A_R agonist binding by several D_2_R agonists, some of them clinically used as antiparkinsonian agents. Apomorphine produced a significantly stronger modulation of A_2A_R agonist binding than pramipexole and rotigotine.^12^

We have recently shown that the canonical G_s_-G_i_ antagonistic interaction, and therefore the functional integrity of the striatal A_2A_R-D_2_R heterotetramer-AC5 complexes, depends on the integrity of very specific interactions between transmembrane domains (TMs) of the receptors, which constitute transmembrane heteromeric and homomeric interfaces, as well as between TMs of the receptors and putative TMs of AC5.^2^ The methodology involved a peptide-interfering approach based on the use of synthetic peptides with the amino acid sequence of all TMs of both receptors and AC5. The synthetic peptides are fused to the HIV transactivator of transcription (TAT), which determines the orientation of the peptide when inserted in the plasma membrane. The TAT-TM peptides are thus screened for their ability to destabilize oligomerization detected by bimolecular fluorescence complementation (BiFC). In this technique, two complementary halves of a fluorescent molecule are separately fused to two different GPCR protomers or to a GPCR protomer and AC5. This peptide-interfering approach is providing a very powerful tool that allows the characterization of all TM interfaces involved in GPCR oligomerization.^2,5,10^

The homomeric TM/TM interactions were the same for both the A_2A_R and the D_2_R homodimers: A TM6/TM6 interface irrespective of cotransfection with the other molecularly different receptors.^2^ The A_2A_R-D_2_R heteromeric interface involved a symmetrical TM4–5/TM5–4 interface, but its integrity also depends on a concomitant strong electrostatic interaction between the C-terminal domain of the A_2A_R (A_2A_R-CT) and the intracellular end of TM5 of the D_2_R. Thus, disruption of the interaction between A_2A_R-CT and D_2_R-TM5 has been shown to significantly reduce A_2A_R-D_2_R heteromerization.^2,10,14–16^ The allosteric interactions between A_2A_R and D_2_R ligands have also been shown to depend on the integrity of the transmembrane and intracellular heteromeric interfaces.^10,14–17^

With its reciprocal canonical and allosteric interactions, the A_2A_R-D_2_R heterotetramer acts as an integrative molecular device, which facilitates the switch between the activation and inhibition of the striatopallidal neuron^7^: A preferential A_2A_R versus D_2_R activation leads to an increase in neuronal activity determined by an A_2A_R-mediated AC5 activation, facilitated by the allosteric counteraction of D_2_R signaling.^7^ Conversely, a preferential D_2_R over A_2A_R activation leads to a decrease in neuronal activity determined by a D_2_R-mediated activation of phospholipase C and facilitated by the canonical and reciprocal allosteric interactions, thus switching off the A_2A_R-mediated AC5 activation.^7^ Indeed, the activation of the striatopallidal neuron leads to withdrawal behaviors and, when intense, to motor arrest and catalepsy. On the contrary, striatopallidal neuron inhibition leads to psychomotor activation.^7,18^

## Functional Role of the Presynaptic Striatal A_1_R-A_2A_R Heterotetramer

Adenosine plays an important modulatory role driving the function of corticostriatal terminals. This control is mediated by adenosine A_1_R and A_2A_R, which establish intermolecular interactions forming A_1_R-A_2A_R heterotetramers.^5,19^ Interestingly, these heteromers work as an adenosine concentration-dependent switch, which determines the opposite effects of adenosine on glutamate release depending on a predominant A_1_R or A_2A_R activation within the heteromer.^19,20^ Importantly, the affinity of adenosine is higher for A_1_R than for A_2A_R. Therefore, low concentrations of adenosine primarily activate A_1_R, which induces an inhibition of glutamate release. On the contrary, on high concentrations of adenosine, such as those obtained under strong glutamatergic transmission and the consequent neuronal and glial ATP release and conversion of ATP on adenosine,^21^ the simultaneous activation of A_2A_R leads to an allosteric modulation within the heteromer, with a reduction of affinity and efficacy of adenosine for the A_1_R.^5,19^ Under these conditions A_2A_R signaling prevails and an adenosine-mediated facilitatory glutamate release is achieved. Importantly, the molecular mechanisms of these interactions are beginning to be understood and they are related to the specific quaternary structure of the A_1_R-A_2A_R heterotetramer.^5^ According to the reciprocal nature of allosterism (see the Functional Role of the Postsynaptic Striatal A_2A_R-D_2_R Heterotetramer section),^13^ we could also expect that A_1_R ligands allosterically modulate A_2A_R ligands in the A_1_R-A_2A_R heterotetramer, but this needs still to be demonstrated.

The A_1_R is a classical G_i_-coupled receptor. However, different to the A_2A_R-D_2_R heterotetramer, the A_1_R-A_2A_R heterotetramer does not sustain a canonical G_s_-G_i_ antagonistic interaction at the AC level and the A_2A_R-mediated AC activation is unopposed by A_1_R activation.^5^ This correlates with the A_2A_R-mediated striatal glutamate release unopposed by A_1_R activation.^19^ Particularly striking was the finding that deletion of A_2A_R-CT enables the canonical G_s_-G_i_ antagonistic interaction in cells coexpressing A_1_R and A_2A_R.^5^ Different from the A_2A_R-D_2_R heterotetramer (see the Functional Role of the Postsynaptic Striatal A_2A_R-D_2_R Heterotetramer section), A_2A_R-CT deletion did not disrupt A_1_R-A_2A_R heteromerization, indicating its lack of participation on the stabilization of the quaternary structure of the A_1_R-A_2A_R heterotetramer.^5^ Interestingly, when compared to the A_2A_R-D_2_R heterotetramer, different TM/TM homomeric and heteromeric interfaces were observed using TAT-TM peptides in BiFC experiments: TM4–5/TM5–4 homomeric interfaces for both A_2A_R and A_1_R homomers and a TM5–6/TM6–5 for the A_2A_R-A_1_R heteromeric interface.^5^ Molecular dynamics computational analysis already predicts that GPCR homodimers display several possible homodimeric TM interfaces.^22^ Our studies indicate that the preferred homomeric interfaces in a GPCR heterotetramer are determined by the heteromeric partner.

An important implication from the study of the role of GPCR heteromers is that they are required for the functional expression of G_i_-coupled receptors in the modulation of certain subtypes of AC. More specifically, those AC isoforms can be inhibited by the α subunits of Gi/o proteins, including AC1, AC5, and AC6.^23^ Artificially, G_i_-coupled receptors can inhibit forskolin-induced AC activation, but we have demonstrated that to inhibit a Gs-coupled receptor-mediated AC5 activation, they need to be part of a GPCR heterotetramer. This has been so far shown for the A_2A_R-D_2_R, A_1_R-D_1_R, and D_1_R-D_3_R heterotetramers, where destabilization of their heteromeric interface leads to the disruption of the canonical G_s_-G_i_ antagonistic interaction at the AC level.^2,24,25^ The A_1_R-A_2A_R heterotetramer represents a particular case, since, because of interference with the A_2A_R-CT, the G_i_-coupled A_1_R cannot counteract the G_s_-coupled A_2A_R-mediated AC activation.^5^ Nevertheless, it is quite well established that the main mechanism involved in the modulation of neurotransmitter release by G_i_-coupled receptors, including A_1_R, is by a decrease in the probability of neurotransmitter release through a direct inhibition of G_i_ protein βγ subunits of N- and P/Q-type voltage-dependent calcium channels.^26,27^ On the contrary, G_s_ protein-coupled receptors can produce and increase in the probability of neurotransmitter release by an AC-cAMP-PKA-dependent mechanism.^28,29^ Therefore, the A_1_R-A_2A_R heterotetramer is particularly designed to inhibit and stimulate glutamate release by an AC-independent and AC-dependent mechanism, respectively.

In addition to providing the frame for allosterical interactions between ligands binding to their orthosteric sites and interactions at the effector level (canonical G_s_-G_i_ antagonistic interaction), heteromerization can lead to changes in the properties of specific ligands for one of the protomers, independent of other ligands binding to the other protomer (Fig. 1C). Indeed, the proof of concept came from experiments in transfected mammalian cells, where the potencies of different selective A_2A_R antagonists at binding to the A_2A_R alone or when coexpressed either with D_2_R or A_1_R were compared.^30^ Interestingly, the most dramatic finding was that the A_2A_R antagonist SCH442416 showed a selective low affinity for the A_2A_R when coexpressed with D_2_R compared with its affinity for the A_2A_R alone or coexpressed with A_1_R (Fig. 1C).^30^ More specifically, SCH442416 showed a pronounced negative cooperativity of its binding to the A_2A_R homodimer within the A_2A_R-D_2_R heterotetramer.^7,30^

This was further demonstrated in striatal preparations from mice with conditional striatal D_2_R-KO, which, in contrast to wild-type mice, did not show the binding negative cooperativity of SCH442416.^7^ As expected, SCH442416 showed a dissociation on its ability to produce locomotor activity (which should depend on its binding to the postsynaptic A_2A_R-D_2_R heterotetramer) versus its ability to inhibit electrically and optogenetically induced striatal glutamate release (which should depend on its binding to the presynaptic A_1_R-A_2A_R heterotetramer).^7,30^ Both in rats and mice, higher systemic doses were necessary to produce locomotion than those necessary to produce inhibition of glutamate release.^7,30^ Importantly, the preferential presynaptic profile of SCH442416 was confirmed by further studies by other research groups^31,32^ and was suggested to provide a therapeutic approach for conditions with increased corticostriatal transmission, such as cannabinoid use disorder.^33^

The same mechanism has recently been reported for the selective significant decrease in potency of the μ-opioid receptor agonist methadone, compared with morphine and fentanyl, in the μ-opioid-galanin Gal_1_ receptor heteromer.^34^ Since these heteromers are selectively localized in the ventral tegmental area (localization of cell bodies of dopaminergic cells involved in the processing of natural rewards), this could explain a selective dissociation of the clinical therapeutic versus euphoric/rewarding effects of methadone compared with other opioids.^34^ Therefore, changes in the pharmacological properties of ligands induced by receptor heteromerization should constitute a very important strategy in the search for new therapeutic agents with further selectivity for their therapeutic versus side, unwanted effects.

## Neuroprotective Role of the Postsynaptic Striatal A_2A_R-D_2_R Heterotetramer: Impact on D_2_R Signaling in PD

PD, the second most common age-related neurodegenerative disorder, is characterized by a progressive loss of nigrostriatal dopaminergic cells, which affects ∼1% of individuals older than 60 years.^35^ PD symptoms include resting tremor, rigidity, bradykinesia, or postural instability and, in advanced stages, cognitive dysfunction and dementia.^36^ Only 10–15% of PD cases classify as early-onset familial PD (recognized as having a first-degree affected family member),^37^ while the remaining cases are idiopathic, thus indicating a key role for nongenetic and environmental factors in PD pathogenesis. Indeed, exposure to environmental toxins (such as pesticides, solvents, heavy metals, and other pollutants) can cause dopaminergic cell death.^38^ Furthermore, oxidative stress, mitochondrial dysfunction, and inflammation play key roles in PD pathopsyciology.^39,40^ Accordingly, PD treatments are mainly based on the use of prodopaminergic drugs, intending to restore neurotransmitter deficiency on the loss of nigrostriatal dopaminergic neurons.^41^ However, this pharmacological treatment also presents many undesired effects, specially on chronic consumption. Thus, although L-DOPA has been proved very efficacious in the treatment of PD symptoms, its long-term treatment tends to lose efficacy and also to induce severe collateral motor effects (dyskinesia and rigidity) and psychiatric symptoms.^42^ Interestingly, most epidemiological studies support a protective benefit of habitual drinking of caffeinated beverages,^43–45^ thus suggesting a neuroprotective role of adenosine receptors in PD.

The striatum plays a central role in PD pathophysiology. Indeed, dopamine depletion throughout the course of PD progression is followed by a substantial cellular and molecular striatal remodeling. Postmortem studies using PD necropsies revealed a reduced dendritic length and spine density in striatal GABAergic neurons.^46^ The caudal part of the putamen, where PD-associated dopamine deficits are more pronounced, shows the highest reduction in spine density.^47^ In addition, some biochemical alterations in postmortem PD brains indicate a protein homeostasis dysregulation,^48^ which includes the expected downregulation of the rate-limiting enzyme in dopamine synthesis tyrosine hydroxylase^49^ and the existence of the intracytoplasmic α-synuclein fibrillar aggregates that constitute the PD histopathological hallmark, the Lewy body.^48^ The levels of several neurotransmitters and the expression of their receptors have been found to be altered, not without some discrepancies.^48^ Thus, while the levels of dopamine are reduced in the whole striatum (caudate, nucleus accumbens, and putamen) and globus pallidus, they were not found to be altered in the subthalamic nucleus, thalamus, and substantia nigra.^50^ Divergences were found when measuring the striatal D_2_R density, with some studies reporting that D_2_R is upregulated,^51^ downregulated,^52^ or unaltered.^53–55^ Interestingly, the adenosinergic system has also been found dysregulated in PD, with no changes in adenosine levels,^56^ but a significant increase in A_2A_R density.^55,57^ Therefore, it is not unreasonable to expect that altered dopamine and adenosine levels and D_2_R and A_2A_R densities within the striatum of PD subjects should determine alterations in the A_2A_R-D_2_R heteromer composition and function, thus impacting PD pathophysiology.

Indeed, we could demonstrate the existence of A_2A_R-D_2_R heteromers in native tissue by using a multimethodological approach (i.e., immunoelectron microscopy, proximity ligation assay, and time-resolved fluorescent resonance energy transfer) in the striatum of control and unilateral 6-OHDA-lesioned rats (a widely used animal model of PD).^58^ Interestingly, a significant reduction of the A_2A_R-D_2_R heteromer content was observed in the 6-OHDA-denervated striatum.^58^ The striatal A_2A_R-D_2_R heteromer disruption observed in this PD animal model might then constitute a neuroadaptive response associated with dopaminergic denervation in PD. Future efforts should determine similar changes in the A_2A_R-D_2_R heteromer status in postmortem caudate-putamen from PD subjects. Indeed, establishing the A_2A_R-D_2_R heteromer status in PD could determine the design of selective combined pharmacotherapeutic strategies restoring the unbalanced A_2A_R-D_2_R heteromer function potentially associated with PD.

In the same PD animal model, the A_2A_R demonstrated a significant constitutive activity and this uncontrolled activity was blocked by caffeine and other prototypic A_2A_R inverse agonists.^59^ Therefore, we assumed that the striatal A_2A_R-D_2_R heteromer disruption observed in this PD animal model, and the expected loss of the negative canonical and allosteric control by the D_2_R was behind the gain of A_2A_R constitutive activity, which could be involved in the striatal neurodegeneration of PD (Fig. 1D). This provides a rationale for the use of A_2A_R antagonists in PD and for fostering the research of mechanisms that could restore an unbalanced expression of A_2A_R homomers versus A_2A_R-D_2_R heteromers in this disease.

## Neuroprotective Role of the Presynaptic Striatal A_1_R-A_2A_R Heterotetramer: Reduction of A_1_R Signaling in RLS

RLS is a very common neurological disorder, characterized by periodic, rest-induced, mostly nocturnal, movement-responsive urge to move the legs or periodic leg movements during sleep (PLMS) and hyperarousal.^60–63^ The deficits of sensorimotor integration that promote PLMS and hyperarousal are interrelated, and adenosine seems to be a very important pathogenetic link. Several preclinical and clinical data indicate the existence of a hypoadenosinergic state secondary to brain iron deficiency (BID) as an initial pathogenetic mechanism in RLS.^63–65^ It has been demonstrated that BID in the experimental animal leads to a generalized downregulation of A_1_R.^66^ A_1_R downregulation in the cortex and in the areas of origin of the ascending arousal systems could explain the hyperarosusal.^63,64^ On the contrary, A_1_R downregulation in the striatum could explain an increased sensitivity of corticostriatal terminals, which has recently proposed to be a main mechanism responsible for the deficits of sensorimotor integration that promote PLMS.^65,67^ In fact, corticostriatal glutamatergic terminals are targets for the drugs most often prescribed in RLS, the dopamine receptor agonists pramipexole and ropinirole and the α_2_δ-ligand gabapentin.^67^

The possible key role of A_1_R downregulation in corticostriatal glutamatergic terminals in the sensorimotor symptomatology of RLS was significantly supported by preclinical and clinical experiments. First, we predicted that equilibrative nucleoside transporter inhibitors, by increasing the striatal extracellular levels of adenosine (which would facilitate the binding probability of adenosine to the lower expressed A_1_R), could provide a new therapeutic approach for RLS. In fact, we recently reported encouraging results with the nonselective ENT1/ENT2 inhibitor dipyridamole in an open trial with RLS patients.^68^ At the preclinical level, as predicted, an A_1_R antagonist produced hypersensitivity of corticostriatal terminals and reduced the frequency of optogenetic stimulation necessary to induce corticostriatal glutamate release.^65^ Furthermore, dipyridamole, by increasing the striatal extracellular concentration of adenosine and the activation of presynaptic A_1_R, was able to counteract optogenetic-induced glutamate release in both naive rats and in rats with BID.^65^ Finally, as we also expected, this effect of dipyridamole was counteracted by the A1R antagonist.^65^

Similar to what seems to occur with postsynaptic A_2A_R in PD, the pathogenesis of RLS could involve a change in the stoichiometry of A_2A_R and A_1_R forming and not forming heterotetramers. A_1_R downregulation would mean a relative increase in A_2A_R/A_1_R expression ratio, which should lead to a relative decrease of A_1_R-A_2A_R heteromers and a relative increase of A_2A_R not forming heteromers. Unopposed by the A_1_R signaling in the heterotetramer, the relative increase in A_2A_R expression and constitutive activity should be indirectly responsible for the A_1_R downregulation-mediated increased sensitivity of the corticostriatal glutamatergic terminals. This would predict that A_2A_R antagonists that target A_2A_R not forming heteromers could also be of therapeutic use in RLS. In fact, we have previously shown that the nonselective pre/postsynaptic A_2A_R antagonist MSX-3 significantly counteracts optogenetically induced corticostriatal glutamate release.^69^

Adenosine A_1_R-A_2A_R heteromers have also been demonstrated in cortical astrocytic cultures, where they modulate GABA transport by GAT-1 and GAT-3 transporters.^70^ The same as the presynaptic striatal A_1_R-A_2A_R heteromer, it acts as an adenosine concentration-dependent switch, which, in this case, determines the opposite effects of adenosine on GABA transport depending on a predominant A_1_R or A_2A_R activation within the heteromer.^70^ If also functionally present in the striatum, it could be involved in the pathogenesis of RLS. A reduction of A_1_R density would imply an increased A_2A_R-mediated GABA transport and, therefore, a reduction in the extracellular levels of the inhibitory neurotransmitter GABA.

## Concluding Remarks

GPCR heteromers are changing classical views of GPCR physiology and pharmacology. First, they are providing a better understanding of interactions between different neurotransmitters and exogenous ligands, based on their ability to convey canonical interactions at the effector level and allosteric interactions between endogenous and exogenous ligands. This, for instance, provides the rationale for the use of A_2A_R antagonists in PD, which increase the therapeutic index of L-DOPA.^71,72^ Second, GPCR heteromerization determines potential pharmacodynamic differences between exogenous compounds, such as the A_2A_R antagonist SCH442416, with its preferential binding to the presynaptic A_1_R-A_2A_R versus postsynaptic A_2A_R-D_2_R heterotetramers. This provides the rationale for the use of SCH442416-like compounds in conditions with an excess of corticostriatal signaling, for instance, in substance use disorders.^31–33,73^ Finally, we believe this review provides sufficient background that supports a pathogenetic role of postsynaptic striatal A_2A_R-D_2_R heterotetramers in PD and presynaptic A_1_R-A_2A_R heterotetramers in RLS, based on their decreased expression versus the expression of the Gs-coupled A_2A_R not forming heteromers, which, free from the antagonistic control of the Gi-coupled D_2_R and A_1_R, facilitate an increased neuronal activation and presynaptic glutamate release, respectively. In both cases, A_2A_R antagonists that could preferentially target A_2A_R not forming heteromers could constitute a successful therapeutic strategy.

## References

[1] Ferré S The GPCR heterotetramer: Challenging classical pharmacology. Trends Pharmacol Sci. 2015;36:145–152 25704194 10.1016/j.tips.2015.01.002 PMC4357316

[2] Navarro G, Cordomí A, Casadó-Anguera V, *et al.* Evidence for functional pre-coupled complexes of receptor heteromers and adenylyl cyclase. Nat Commun. 2018;9:1242 29593213 10.1038/s41467-018-03522-3 PMC5871782

[3] Ferré S, Casadó V, Devi LA, *et al.* G protein-coupled receptor oligomerization revisited: Functional and pharmacological perspectives. Pharmacol Rev. 2014;66:413–434 24515647 10.1124/pr.113.008052 PMC3973609

[4] Ferré S, Baler R, Bouvier M, *et al.* Building a new conceptual framework for receptor heteromers. Nat Chem Biol. 2009;5:131–134 19219011 10.1038/nchembio0309-131 PMC2681085

[5] Navarro G, Cordomí A, Brugarolas M, *et al.* Cross-communication between G(i) and G(s) in a G-protein-coupled receptor heterotetramer guided by a receptor C-terminal domain. BMC Biol. 2018;16:24 29486745 10.1186/s12915-018-0491-x PMC6389107

[6] Gerfen CR Basal ganglia. In: The Rat Nervous System. Paxinos G (Ed). Amsterdam: Elsevier; 2004: pp. 445–508

[7] Ferré S, Bonaventura J, Zhu W, *et al.* Essential control of the function of the striatopallidal neuron by pre-coupled complexes of adenosine A(2A)-dopamine D(2) receptor heterotetramers and adenylyl cyclase. Front Pharmacol. 2018;9:243 29686613 10.3389/fphar.2018.00243 PMC5900444

[8] Gilman AG G proteins: Transducers of receptor-generated signals. Annu Rev Biochem. 1987;56:615–649 3113327 10.1146/annurev.bi.56.070187.003151

[9] Dessauer CW, Tesmer JJ, Sprang SR, *et al.* Identification of a Gialpha binding site on type V adenylyl cyclase. J Biol Chem. 1998;273:25831–25839 9748257 10.1074/jbc.273.40.25831

[10] Bonaventura J, Navarro G, Casadó-Anguera V, *et al.* Allosteric interactions between agonists and antagonists within the adenosine A2A receptor-dopamine D2 receptor heterotetramer. Proc Natl Acad Sci U S A. 2015;112:E3609–E3618 26100888 10.1073/pnas.1507704112 PMC4500251

[11] Fernández-Dueñas V, Gómez-Soler M, Jacobson KA, *et al.* Molecular determinants of A2AR-D2R allosterism: Role of the intracellular loop 3 of the D2R. J Neurochem. 2012;123:373–384 22924752 10.1111/j.1471-4159.2012.07956.x PMC3480334

[12] Fernández-Dueñas V, Gómez-Soler M, Morató X, *et al.* Dopamine D(2) receptor-mediated modulation of adenosine A(2A) receptor agonist binding within the A(2A)R/D(2)R oligomer framework. Neurochem Int. 2013;63:42–46 23619397 10.1016/j.neuint.2013.04.006 PMC3705641

[13] Kenakin TP Biased signalling and allosteric machines: New vistas and challenges for drug discovery. Br J Pharmacol. 2012;165:1659–1669 22023017 10.1111/j.1476-5381.2011.01749.x PMC3372820

[14] Ciruela F, Burgueño J, Casadó V, *et al.* Combining mass spectrometry and pull-down techniques for the study of receptor heteromerization. Direct epitope-epitope electrostatic interactions between adenosine A2A and dopamine D2 receptors. Anal Chem. 2004;76:5354–5363 15362892 10.1021/ac049295f

[15] Borroto-Escuela DO, Romero-Fernandez W, Tarakanov AO, *et al.* Characterization of the A2AR-D2R interface: Focus on the role of the C-terminal tail and the transmembrane helices. Biochem Biophys Res Commun. 2010;402:801–807 21040702 10.1016/j.bbrc.2010.10.122

[16] Borroto-Escuela DO, Marcellino D, Narvaez M, *et al.* A serine point mutation in the adenosine A2AR C-terminal tail reduces receptor heteromerization and allosteric modulation of the dopamine D2R. Biochem Biophys Res Commun. 2010;394:222–227 20197060 10.1016/j.bbrc.2010.02.168

[17] Navarro G, Ferré S, Cordomi A, *et al.* Interactions between intracellular domains as key determinants of the quaternary structure and function of receptor heteromers. J Biol Chem. 2010;285:27346–27359 20562103 10.1074/jbc.M110.115634 PMC2930733

[18] Taura J, Valle-León M, Sahlholm K, *et al.* Behavioral control by striatal adenosine A(2A)-dopamine D(2) receptor heteromers. Genes Brain Behav. 2018;17:e12432 29053217 10.1111/gbb.12432

[19] Ciruela F, Casadó V, Rodrigues RJ, *et al.* Presynaptic control of striatal glutamatergic neurotransmission by adenosine A1-A2A receptor heteromers. J Neurosci. 2006;26:2080–2087 16481441 10.1523/JNEUROSCI.3574-05.2006 PMC6674939

[20] Ciruela F, Ferré S, Casadó V, *et al.* Heterodimeric adenosine receptors: A device to regulate neurotransmitter release. Cell Mol Life Sci. 2006;63:2427–2431 17058035 10.1007/s00018-006-6216-2 PMC11136455

[21] Cunha RA How does adenosine control neuronal dysfunction and neurodegeneration? J Neurochem. 2016;139:1019–1055 27365148 10.1111/jnc.13724

[22] Provasi D, Boz MB, Johnston JM, Filizola M Preferred supramolecular organization and dimer interfaces of opioid receptors from simulated self-association. PLoS Comput Biol. 2015;11:e1004148 25822938 10.1371/journal.pcbi.1004148 PMC4379167

[23] Sadana R, Dessauer CW Physiological roles for G protein-regulated adenylyl cyclase isoforms: Insights from knockout and overexpression studies. Neurosignals. 2009;17:5–22 18948702 10.1159/000166277 PMC2790773

[24] Rivera-Oliver M, Moreno E, Álvarez-Bagnarol Y, *et al.* Adenosine A(1)-dopamine D(1) receptor heteromers control the excitability of the spinal motoneuron. Mol Neurobiol. 2019;56:797–811 29797183 10.1007/s12035-018-1120-y PMC6252157

[25] Guitart X, Moreno E, Rea W, *et al* Biased G protein-independent signaling of dopamine D(1)-D(3) receptor heteromers in the nucleus accumbens. Mol Neurobiol. 2019 [Epub ahead of print]; DOI: 10.1007/s12035-019-1564-8 PMC6728209 30919214

[26] Wu LG, Saggau P Presynaptic inhibition of elicited neurotransmitter release. Trends Neurosci. 1997;20:204–212 9141196 10.1016/s0166-2236(96)01015-6

[27] Jarvis SE, Zamponi GW Interactions between presynaptic Ca2+ channels, cytoplasmic messengers and proteins of the synaptic vesicle release complex. Trends Pharmacol Sci. 2001;22:519–525 11583809 10.1016/s0165-6147(00)01800-9

[28] Evans GJ, Morgan A Regulation of the exocytotic machinery by cAMP-dependent protein kinase: Implications for presynaptic plasticity. Biochem Soc Trans. 2003;31:824–827 12887314 10.1042/bst0310824

[29] Leenders AG, Sheng ZH Modulation of neurotransmitter release by the second messenger-activated protein kinases: Implications for presynaptic plasticity. Pharmacol Ther. 2005;105:69–84 15626456 10.1016/j.pharmthera.2004.10.012 PMC1804289

[30] Orru M, Bakešová J, Brugarolas M, *et al.* Striatal pre- and postsynaptic profile of adenosine A(2A) receptor antagonists. PLoS One. 2011;6:e16088 21264319 10.1371/journal.pone.0016088 PMC3019225

[31] O'Neill CE, Hobson BD, Levis SC, *et al.* Persistent reduction of cocaine seeking by pharmacological manipulation of adenosine A1 and A 2A receptors during extinction training in rats. Psychopharmacology. 2014;231:3179–3188 24562064 10.1007/s00213-014-3489-2 PMC4111968

[32] Haynes NS, O'Neill CE, Hobson BD, *et al.* Effects of adenosine A(2A) receptor antagonists on cocaine-induced locomotion and cocaine seeking. Psychopharmacology (Berl). 2019;236:699–708 30392131 10.1007/s00213-018-5097-z PMC6401288

[33] Justinová Z, Redhi GH, Goldberg SR, *et al.* Differential effects of presynaptic versus postsynaptic adenosine A2A receptor blockade on Δ9-tetrahydrocannabinol (THC) self-administration in squirrel monkeys. J Neurosci. 2014;34:6480–6484 24806674 10.1523/JNEUROSCI.5073-13.2014 PMC4012307

[34] Cai NS, Quiroz C, Bonaventura J, *et al.* Opioid-galanin receptor heteromers mediate the dopaminergic effects of opioids. J Clin Invest. 2019;130:126912 10.1172/JCI126912 PMC6597217 30913037

[35] Ascherio A, Schwarzschild MA The epidemiology of Parkinson's disease: Risk factors and prevention. Lancet Neurol. 2016;15:1257–1272 27751556 10.1016/S1474-4422(16)30230-7

[36] de Lau LM, Breteler MM Epidemiology of Parkinson's disease. Lancet Neurol. 2006;5:525–535 16713924 10.1016/S1474-4422(06)70471-9

[37] Karimi-Moghadam A, Charsouei S, Bell B, *et al.* Parkinson disease from mendelian forms to genetic susceptibility: New molecular insights into the neurodegeneration process. Cell Mol Neurobiol. 2018;38:1153–1178 29700661 10.1007/s10571-018-0587-4 PMC6061130

[38] Goldman SM Environmental toxins and Parkinson's disease. Annu Rev Pharmacol Toxicol. 2014;54:141–164 24050700 10.1146/annurev-pharmtox-011613-135937

[39] Gelders G, Baekelandt V, Van der Perren A Linking neuroinflammation and neurodegeneration in Parkinson's disease. J Immunol Res. 2018;2018:4784268 29850629 10.1155/2018/4784268 PMC5926497

[40] Grünewald A, Kumar KR, Sue CM New insights into the complex role of mitochondria in Parkinson's disease. Prog Neurobiol. 2019;177:73–93 30219247 10.1016/j.pneurobio.2018.09.003

[41] Meissner WG, Frasier M, Gasser T, *et al.* Priorities in Parkinson's disease research. Nat Rev Drug Discov. 2011;10:377–393 21532567 10.1038/nrd3430

[42] Huot P, Johnston TH, Koprich JB, *et al.* The pharmacology of L-DOPA-induced dyskinesia in Parkinson's disease. Pharmacol Rev. 2013;65:171–222 23319549 10.1124/pr.111.005678

[43] Ascherio A, Zhang SM, Hernán MA, *et al.* Prospective study of caffeine consumption and risk of Parkinson's disease in men and women. Ann Neurol. 2001;50:56–63 11456310 10.1002/ana.1052

[44] Ross GW, Abbott RD, Petrovitch H, *et al.* Association of coffee and caffeine intake with the risk of Parkinson disease. JAMA. 2000;283:2674–2679 10819950 10.1001/jama.283.20.2674

[45] Sääksjärvi K, Knekt P, Rissanen H, *et al.* Prospective study of coffee consumption and risk of Parkinson's disease. Eur J Clin Nutr. 2008;62:908–915 17522612 10.1038/sj.ejcn.1602788

[46] Stephens B, Mueller AJ, Shering AF, *et al.* Evidence of a breakdown of corticostriatal connections in Parkinson's disease. Neuroscience. 2005;132:741–754 15837135 10.1016/j.neuroscience.2005.01.007

[47] Zaja-Milatovic S, Milatovic D, Schantz AM, *et al.* Dendritic degeneration in neostriatal medium spiny neurons in Parkinson disease. Neurology. 2005;64:545–547 15699393 10.1212/01.WNL.0000150591.33787.A4

[48] Toulorge D, Schapira AH, Hajj R Molecular changes in the postmortem parkinsonian brain. J Neurochem. 2016;139 Suppl 1:27–58 27381749 10.1111/jnc.13696

[49] Ryoo HL, Pierrotti D, Joyce JN Dopamine D3 receptor is decreased and D2 receptor is elevated in the striatum of Parkinson's disease. Mov Disord. 1998;13:788–797 9756147 10.1002/mds.870130506

[50] Gerlach M, Gsell W, Kornhuber J, *et al.* A post mortem study on neurochemical markers of dopaminergic, GABA-ergic and glutamatergic neurons in basal ganglia-thalamocortical circuits in Parkinson syndrome. Brain Res. 1996;741:142–152 9001716 10.1016/s0006-8993(96)00915-8

[51] Piggott MA, Marshall EF, Thomas N, *et al.* Striatal dopaminergic markers in dementia with Lewy bodies, Alzheimer's and Parkinson's diseases: Rostrocaudal distribution. Brain. 1999;122:1449–1468 10430831 10.1093/brain/122.8.1449

[52] Ahlskog JE, Richelson E, Nelson A, *et al.* Reduced D2 dopamine and muscarinic cholinergic receptor densities in caudate specimens from fluctuating parkinsonian patients. Ann Neurol. 1991;30:185–191 1654766 10.1002/ana.410300210

[53] Griffiths PD, Perry RH, Crossman AR A detailed anatomical analysis of neurotransmitter receptors in the putamen and caudate in Parkinson's disease and Alzheimer's disease. Neurosci Lett. 1994;169:68–72 8047295 10.1016/0304-3940(94)90358-1

[54] Mattila PM, Röyttä M, Lönnberg P, *et al.* Choline acetytransferase activity and striatal dopamine receptors in Parkinson's disease in relation to cognitive impairment. Acta Neuropathol. 2001;102:160–166 11563631 10.1007/s004010100372

[55] Varani K, Vincenzi F, Tosi A, *et al.* A2A adenosine receptor overexpression and functionality, as well as TNF-alpha levels, correlate with motor symptoms in Parkinson's disease. FASEB J. 2010;24:587–598 19776336 10.1096/fj.09-141044

[56] McFarland NR, Burdett T, Desjardins CA, *et al.* Postmortem brain levels of urate and precursors in Parkinson's disease and related disorders. Neurodegener Dis. 2013;12:189–198 23467193 10.1159/000346370 PMC3809155

[57] Villar-Menéndez I, Porta S, Buira SP, *et al.* Increased striatal adenosine A2A receptor levels is an early event in Parkinson's disease-related pathology and it is potentially regulated by miR-34b. Neurobiol Dis. 2014;69:206–214 24892887 10.1016/j.nbd.2014.05.030

[58] Fernández-Dueñas V, Taura JJ, Cottet M, *et al.* Untangling dopamine-adenosine receptor-receptor assembly in experimental parkinsonism in rats. Dis Model Mech. 2015;8:57–63 25398851 10.1242/dmm.018143 PMC4283650

[59] Fernández-Dueñas V, Gómez-Soler M, López-Cano M, *et al.* Uncovering caffeine's adenosine A2A receptor inverse agonism in experimental parkinsonism. ACS Chem Biol. 2014;9:2496–2501 25268872 10.1021/cb5005383 PMC4245165

[60] Allen RP, Walters AS, Montplaisir J, *et al.* Restless legs syndrome prevalence and impact: REST general population study. Arch Intern Med. 2005;165:1286–1292 15956009 10.1001/archinte.165.11.1286

[61] Allen RP, Stillman P, Myers AJ Physician-diagnosed restless legs syndrome in a large sample of primary medical care patients in western Europe: Prevalence and characteristics. Sleep Med. 2010;11:31–37 19464949 10.1016/j.sleep.2009.03.007

[62] Ferri R, Rundo F, Zucconi M, *et al.* An evidence-based analysis of the association between periodic leg movements during sleep and arousals in Restless Legs Syndrome. Sleep. 2015;38:919–924 25581922 10.5665/sleep.4740 PMC4434558

[63] Ferré S, García-Borreguero D, Allen RP, *et al.* New insights into the neurobiology of Restless Legs Syndrome. Neuroscientist. 2019;25:113–125 30047288 10.1177/1073858418791763 PMC9372713

[64] Ferré S, Quiroz C, Guitart X, *et al.* Pivotal role of adenosine neurotransmission in restless legs syndrome. Front Neurosci. 2018;11:722 29358902 10.3389/fnins.2017.00722 PMC5766678

[65] Ferré S, Quiroz C, Rea W, *et al.* Adenosine mechanisms and hypersensitive corticostriatal terminals in restless legs syndrome. Rationale for the use of inhibitors of adenosine transport. Adv Pharmacol. 2019;84:3–19 31229176 10.1016/bs.apha.2018.12.005 PMC9372712

[66] Quiroz C, Gulyani S, Ruiqian W, *et al.* Adenosine receptors as markers of brain iron deficiency: Implications for Restless Legs Syndrome. Neuropharmacology. 2016;111:160–168 27600688 10.1016/j.neuropharm.2016.09.002 PMC5056844

[67] Yepes G, Guitart X, Rea W, *et al.* Targeting hypersensitive corticostriatal terminals in restless legs syndrome. Ann Neurol. 2017;82:951–960 29171915 10.1002/ana.25104 PMC5739944

[68] García-Borreguero D, Guitart X, García Malo C, *et al.* Treatment of restless legs syndrome/Willis-Ekbom disease with the non-selective ENT1/ENT2 inhibitor dipyridamole: Testing the adenosine hypothesis. Sleep Med. 2018;45:94–97 29680437 10.1016/j.sleep.2018.02.002

[69] Quiroz C, Orrú M, Rea W, *et al.* Local control of extracellular dopamine levels in the medial nucleus accumbens by a glutamatergic projection from the infralimbic cortex. J Neurosci. 2016;36:851–859 26791215 10.1523/JNEUROSCI.2850-15.2016 PMC4719020

[70] Cristóvão-Ferreira S, Navarro G, Brugarolas M, *et al.* A1R-A2AR heteromers coupled to Gs and G i/0 proteins modulate GABA transport into astrocytes. Purinergic Signal. 2013;9:433–449 23657626 10.1007/s11302-013-9364-5 PMC3757138

[71] Jenner P Istradefylline, a novel adenosine A2A receptor antagonist, for the treatment of Parkinson's disease. Expert Opin Investig Drugs. 2005;14:729–738 10.1517/13543784.14.6.729 16004599

[72] Armentero MT, Pinna A, Ferré S, *et al.* Past, present and future of A(2A) adenosine receptor antagonists in the therapy of Parkinson's disease. Pharmacol Ther. 2011;132:280–299 21810444 10.1016/j.pharmthera.2011.07.004 PMC3205226

[73] Kravitz AV, Tomasi D, LeBlanc KH, *et al.* Cortico-striatal circuits: Novel therapeutic targets for substance use disorders. Brain Res. 2015;1628:186–198 25863130 10.1016/j.brainres.2015.03.048 PMC9364041

